# *Notes from the Field:* Coagulopathy Associated with Brodifacoum Poisoning — Florida, December 2021

**DOI:** 10.15585/mmwr.mm7140a5

**Published:** 2022-10-07

**Authors:** Nikki Coble, Prakash Mulay, Alexandra Funk, Justin Arnold, Michael Wiese

**Affiliations:** ^1^Florida Department of Health Hillsborough County, Tampa, Florida; ^2^Florida Department of Health; ^3^Florida Poison Information Center Tampa, Tampa, Florida.

On December 4, 2021, the Florida Department of Health in Hillsborough County was notified by the Florida Poison Information Center Tampa about three patients with unexplained bleeding and a history of synthetic cannabinoid (SCB) use. These patients resembled those from the nationwide incident of coagulopathy associated with SCB use that occurred in 2018, which included five patients from Florida who displayed similar signs, symptoms, and high-risk behaviors (*1*). An epidemiologic investigation was conducted to establish exposure links and provide guidance to hospitals and health care providers. On December 7, 2021, epidemiology program managers at county health departments in the region including Pasco County, Pinellas County, and Polk County, and emergency department physicians as well as medical examiners at Advent Health, St. Joseph Hospital, and Tampa General Hospital were informed about these three patients and asked to report any suspected cases. A press release was issued to the public for awareness. Florida’s syndromic surveillance database, Electronic Surveillance System for the Early Notification of Community-based Epidemics, was used to monitor Florida Poison Information Center, emergency department, and urgent care data for potential new cases. Case definitions were established based on the nationwide 2018 incident (*1*). Patients were interviewed, and medical records were reviewed to collect information on patient demographics; signs and symptoms; SCB, marijuana, or other drug use; product purchase locations; and exposure to prescription vitamin K oxidoreductase antagonists.

A total of 52 cases were identified; 43 (82.7%) were confirmed and nine (17.3%) probable. A total of 38 (73.1%) cases were distributed throughout north and east Tampa; the other cases occurred sporadically throughout Hillsborough County. One patient was identified in neighboring Pinellas County. All patients except one were admitted to hospitals in Hillsborough County. The mean patient age was 36 years (range = 16–63 years); 40 (76.9%) were male. A total of 47 (87.0%) reported using SCBs with similar purchase locations before symptom onset. Five patients had both elevated international normalized ratios (INRs) and positive brodifacoum tests but did not report SCB use.* Symptom onset occurred during November 24–December 19, 2021 (Figure). The most common symptoms were hematuria (36; 69.2%), abdominal pain (33; 63.5%), and hematemesis (16; 30.8%). INR measurements were elevated in all patients; the median INR was 12.8 (range = 3.9 to >15) (*2*). Four (7.7%) patients died; the mean age of deceased patients was 34 years.

**FIGURE:**
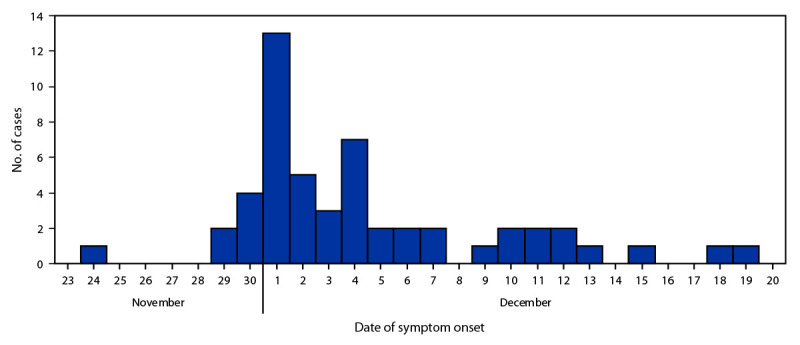
Cases* of coagulopathy associated with brodifacoum poisoning, by date of symptom onset — Florida, November–December 2021 * Total number of cases = 52.

None of the patients reported taking prescribed vitamin K oxidoreductase antagonists that could cause a substantial change in INR measurements. Five patients provided the SCB products they had smoked, of which four tested positive for brodifacoum, a long-acting vitamin K oxidoreductase antagonist.^†^ All five products tested positive for the SCBs 4F-MDMB-BUTICA and ADB-BUTINACA.

Vitamin K1 was used to treat vitamin K oxidoreductase antagonist coagulopathy; treatment was administered by both oral and intravenous routes. Patients began with intravenous vitamin K1 and transitioned to oral vitamin K1. Many patients needed high doses of oral vitamin K1 (i.e., 150 mg/day), which required taking 30 5-mg tablets daily during hospitalization and for 3–6 months after discharge, with treatment time varying for each patient based on their brodifacoum terminal elimination (*3*). Approximately two thirds of patients (34; 65.4%) were uninsured and unable to pay for inpatient and outpatient treatment; oral vitamin K1 treatment can cost ≥$65,000 per month. With assistance from Hillsborough County, 12 patients were enrolled in a local managed health care program for residents with limited income. A private pharmaceutical company donated enough vitamin K1 tablets to treat all 52 patients.

Three major challenges were identified during this incident response. First, diagnosis of a specific vitamin K oxidoreductase antagonist (i.e., brodifacoum) was challenging because diagnosis required testing against an anticoagulant panel that is expensive (i.e., >$750 per specimen), has long turnaround time, and is only offered by a single private laboratory. Initially, this laboratory performed only qualitative analysis while the testing to be able to perform quantitative brodifacoum testing was calibrated. Once the qualitative result was positive, the laboratory performed quantitative brodifacoum testing to aid in patient monitoring throughout the event. Through discussion with Florida Poison Information Center Tampa, the private laboratory was able to reduce the cost of quantitative brodifacoum testing and decrease turnaround time for patients involved in this event. Serial quantitative brodifacoum testing was eventually performed to help determine when therapy could be discontinued (*3*). Second, treatment required high doses of vitamin K1 during an extended period of time, and local pharmacies only had a limited supply.^§^ Before the private pharmaceutical company donated the vitamin K1 tablets, a contingency plan was developed to obtain them from other hospitals in the region in the event pharmacies were to run out of supply. Third, maintaining patient compliance and adherence to this treatment plan is challenging because of the high cost and cumbersome treatment regimen (*4*). These challenges reflect similar issues that arose during the 2018 incident; all stakeholders should discuss these issues and identify solutions for optimal patient care.

Communicating timely information to health care providers and the general public allowed for additional patient identification and was crucial to connecting with patients who needed medical care. Close collaboration among the health care community, Florida Department of Health, Florida Poison Information Center Tampa, laboratories, a private pharmaceutical company, and other stakeholders, such as local law enforcement and the Drug Enforcement Agency, was critical to identifying and characterizing the cluster and providing the necessary treatment to prevent additional morbidity and mortality. To help avert future distribution of brodifacoum-laced SCB products, local county law enforcement was informed of this incident and provided information regarding the locations where patients reported they had purchased SCB products.

## Footnotes

* INR is a laboratory measurement of how long blood takes to form a clot. INR is used to determine the effects of oral anticoagulants on the clotting system.

† Brodifacoum is a vitamin K epoxide cycle antagonist with a long half-life resulting in prolonged symptoms and treatment. Clinical coagulopathy is caused by a depletion of functional (vitamin K-dependent) clotting factors as a result of vitamin K oxidoreductase inhibition caused by brodifacoum.

§ In addition to oral vitamin K1, treatment with cholestyramine was also considered. However, this treatment was not used because of concerns regarding patient compliance to take several medications timed appropriately and potential adverse events from long-term cholestyramine use. Cholestyramine can affect oral absorption of vitamin K1 if taken concurrently, which would reduce absorption of oral vitamin K1 for therapeutic purposes.

## References

[1] Moritz E, Austin C, Wahl M, Notes from the field: outbreak of severe illness linked to the vitamin K antagonist brodifacoum and use of synthetic cannabinoids—Illinois, March–April 2018. MMWR Morb Mortal Wkly Rep 2018;67:607–8. 10.15585/mmwr.mm6721a4 29851941 PMC6038901

[2] Riley RS, Rowe D, Fisher LM. Clinical utilization of the international normalized ratio (INR). J Clin Lab Anal 2000;14:101–14. 10.1002/(SICI)1098-2825(2000)14:3<101::AID-JCLA4>3.0.CO;2-A 10797608 PMC6807747

[3] Yip L, Stanton NV, Middleberg RA. Vitamin K1 treatment duration in patients with brodifacoum poisoning. N Engl J Med 2020;382:1764–5. 10.1056/NEJMc1916199 32348649

[4] Tole M, LaBedz S, Feinstein DL, Rubinstein I. Adherence to long-term follow-up of patients with life-threatening, inhaled synthetic cannabinoids-associated coagulopathy in Chicago. Lung 2019;197:349–52. 10.1007/s00408-019-00227-2 31004190 PMC6522275

