# *Erratum:* Vol. 71, No 40

**DOI:** 10.15585/mmwr.mm7150a6

**Published:** 2022-12-16

In the report, “Notes from the Field: Coagulopathy Associated with Brodifacoum Poisoning 2014 — Florida, December 2021,” on page 1288, the second sentence of the third paragraph should have read, “Five patients provided the SCB products they had smoked **for analysis by the DEA TOX Toxicology Testing Program,** of which four tested positive for brodifacoum, a long-acting vitamin K oxidoreductase antagonist.^†”^ On page 1289, the second sentence of the first paragraph should have read, “Close collaboration among the health care community, Florida Department of Health, Florida Poison Information Center Tampa, **DEA TOX, NMS Labs, and a private pharmaceutical company,** **in addition to** other stakeholders such as local law enforcement and the Drug Enforcement Agency, was critical to identifying and characterizing the cluster and providing the necessary treatment to prevent additional morbidity and mortality.” In addition, on page 1289, the Acknowledgments should have included **“Roy Gerona, Jordan Trecki, DEA TOX Toxicology Testing Program.”**

